# Cognitive load affects gaze dynamics during real-world tasks

**DOI:** 10.1007/s00221-025-07037-4

**Published:** 2025-03-03

**Authors:** A. P. Martinez-Cedillo, N. Gavrila, A. Mishra, E. Geangu, T. Foulsham

**Affiliations:** 1 Department of Psychology, University of York, York, YO10 5DD UK; 2 Department of Psychology, University of Essex, Wivenhoe Park, Colchester, Essex, CO4 3SQ UK

**Keywords:** Eye movement, Natural tasks, Sequential, Cognitive Load

## Abstract

In everyday tasks, active gaze is used to gather information for the actions we perform. The cognitive resources required for such gaze control have rarely been investigated. We examined how a secondary cognitive load task would affect gaze during tea- and sandwich-making, everyday tasks which involve sequences of object-related actions (Hayhoe in Vis Cogn 7(1–3):43–64, 2000 and Land et al. in Perception 28(11):1311–1328, 1999). Participants performed these tasks while wearing a mobile eye-tracker, while also counting backwards by threes (high cognitive load) or by ones (low cognitive load). Our findings revealed that participants were slower in tasks and sub-tasks and exhibited more fixations on irrelevant objects in high-load than low-load conditions. Furthermore, the eye-hand span was reduced under high-load conditions, meaning that participants were less likely to look ahead of their manual actions. These findings reveal specific effects of cognitive load in realistic, everyday situations, and begin to shed light on the mechanisms behind gaze control in active tasks. These mechanisms are not resource-free.

## Introduction

Performing a visual task requires acquiring the necessary information at the right time. In everyday life, humans accomplish this by moving their gaze around the environment. The processes involved in guiding gaze have often been overlooked in accounts of everyday behaviour, where the tasks are often more complex, and maintaining effective gaze control must require cognitive resources. In the present study we investigate the deployment of these resources by manipulating cognitive load in a realistic task.

Several studies have investigated the role of gaze in performing everyday tasks, including walking, searching for an object, playing the piano, making tea or sandwiches, and playing sports (Ballard and Hayhoe 2009; Kothari et al. 2020; Land and Tatler 2009). Through these studies, researchers have found that gaze patterns are quite specific to each task, and that they are highly targeted towards relevant objects, helping us to perform efficiently. For example, recent analysis of gaze whilst walking shows how participants fixate the ground a few steps in advance of treading on a particular location (Matthis et al. 2018; 2022). When terrain is uneven, participants spend more time looking at the ground near to their feet. Gaze is therefore adapted to extract information according to what is needed for planning footsteps, helping walkers balance and navigate the terrain.

Our study examines gaze during two everyday tasks: making a cup of tea and making a sandwich. These tasks have been the focus of a number of investigations in the past 30 years (Foulsham 2015; Hayhoe et al. 2012; Land, and Hayhoe 2001; Land and Tatler 2009; Land et al. 1999). Land and Hayhoe (2001) break down these tasks into sequences of object-related actions (ORA), which refers to all the actions performed on a particular object without interruption. It was found that participants were quite consistent in the way they focused on the most relevant objects at a particular time relative to the key ORAs. For example, before reaching for an object, participants tended to fixate that object around half to one second before making contact with the hand. In other cases, participants made “look-ahead fixations” further in advance, suggesting that future targets were being planned in advance and memorised. This type of descriptive analysis has continued to be applied to activities that require coordination of eyes, limbs and body, such as assembling a camping tent (Sullivan et al. 2021).

Tasks such as tea-making have also been of considerable interest for cognitive neuropsychology because they are particularly sensitive to disruptions in cognitive abilities such as attention and planning (Forde and Humphreys 2000; Ward and Morris 2004). Allain et al. (2014) asked older adults with Alzheimer's, and a control group, to make a cup of coffee. Despite prior training, Alzheimer's patients made more errors and took longer to complete the task than the control group. Other studies have compared patients with right- and left-sided brain damage and described differences in the action sequence plan, suggesting compensation in how patients complete the tea-making task (Bieńkiewicz, et al. 2015). Research by Forde et al. (2010) added the measurement of gaze position to neuropsychological assessment of everyday tasks. Their studies investigated gaze behaviour during tea-making with two patients with action disorganization syndrome (ADS). In this syndrome, participants have difficulty carrying out tasks in which multiple actions must be carried out in order, which had been previously attributed to a motor deficit (Schwartz et al. 1991). However, the gaze patterns of these patients revealed a more subtle impairment. Both patients tended to look at more irrelevant than relevant objects, suggesting an attentional impairment, and they often lacked look-ahead fixations, revealing a deficit in planning ahead.

Morady and Humphreys (2009) evaluated the mistakes made by a patient with ADS (Experiment 1) in various everyday tasks, and in conditions where sometimes other distractor objects were present. The ADS patient made more errors when distractors were present. In a second experiment, more action errors were made when healthy control participants had to concurrently perform a more difficult task (requiring manipulating numbers in memory, compared to an easier task where they simply had to respond with the word “the” when prompted). There were no effects of the relatedness of the distracting objects on action errors. However, this study did not monitor gaze, which could enable researchers to detect more subtle variations with load, even among unimpaired individuals.

The results from neuropsychology suggest that gaze control may be affected by distraction under conditions of reduced cognitive capacity. However, the cognitive resources required for the specific types of gaze behaviour observed in active tasks remains unknown. In laboratory tasks, the process of attending to a visual target (and ignoring distractors) is affected by the availability of working memory resources. For example, goal-based attention is disrupted when participants must perform a concurrent task such as remembering a number or counting backwards (Boot et al. 2005; Burnham et al. 2014; Lavie 2010; Lavie et al. 2004, 2005). This interference results in increased distraction (e.g., larger compatibility effects in the flanker task; Lavie et al 2004; Martinez-Cedillo et al 2022, Experiment 1). The load theory of attention explains these effects, linking working memory resources to the efficiency with which attention can select information. In recent studies, we found that a verbal working memory load (remembering a number) did not have an effect on eye movements while freely viewing a picture, although this may depend on the type of load task (Martinez-Cedillo et al 2024).

Previous studies have investigated the potential impact of a secondary task on gaze behaviour, but these have typically not considered the cognitive load implied by the secondary task, or the sequential actions which are critical in everyday tasks. For instance, Jovancevic et al., (2006) asked participants to navigate a virtual environment with other pedestrians who had to be avoided. Adding a dual task which required looking elsewhere in the environment (following a leader) reduced fixations on potential collisions. Zukowski et al. (2021) instead used a cognitive, category-naming dual task. Participants walked for 1 min either without distraction or while performing the category-naming task at the same time. The dual task led to slower walking, but it also changed gaze behaviour, with participants focusing more on the near path. Interestingly, other studies of walking in the presence of a dual task have not found effects on gaze behaviour. Walsh and Snowball (2023) report no effect of cognitive or visual dual tasks on gaze during treadmill walking. Müller et al. (2023), meanwhile, find that a cognitive dual task (counting backwards in sevens) affects gait during walking, and leads to a different pattern of head movements (indicative of looking more at the sky while completing the cognitive task). Thus, the specific effects of a dual task on walking appear quite mixed, and there are, to our knowledge, no studies of the effect of load on gaze in sequential tasks such as tea making. In laboratory tasks using gaze, dual task paradigms tend to result in slower eye movements, reflecting response interference (e.g., Lamers and Roelofs 2011). We would therefore expect realistic tasks to be delayed by a challenging dual task, but the source of this delay, and the specific aspects of eye movement control which are affected, remain unknown.

### Present research

In the present study, we address the question of how cognitive load affects gaze in healthy participants carrying out everyday tasks. The participants were instructed to count backwards either by threes (high-load) or ones (low-load) while making a cup of tea and a sandwich.

Individuals engaged in the higher-load dual task would be expected to perform more slowly. We hypothesised that if attending efficiently in this context requires cognitive resources, there should also be evidence of increased distraction in high load (vs low load), such as increased looking at non-relevant objects. If all or some aspects of gaze control remain unaffected by the amount of load, then it would instead suggest that individuals draw on resources separate from other sorts of visuospatial attention.

## Materials and methods

### Participants

Twenty-five healthy adults participated (ages 18 – 59, *M* = *27.80, SD* = *8.43 years*, 18 females; 7 males). All participants reported normal or corrected-to-normal vision and were paid £5. For full transparency, our experiment's pre-registration form, data, experimental details, and analysis code are accessible on the Open Science Framework at: https://osf.io/6qnwj/?view_only=8d16cc94859845b89f8370a13219f7c7

### Apparatus

Eye movements were recorded using the Positive Science head-mounted eye tracker (www.positivescience.com) at a sampling rate averaging 30 Hz. The headset included an infrared LED, an eye camera for monocular gaze tracking, and a scene camera fitted with a wide-angle lens with a field-of-view of approximately 82 × 68 degrees. Scene recordings were captured at 30 fps (variable) and 640 × 480 resolution.

### Procedure

The experiment took place in a lab environment designed to resemble a domestic setting. After participants gave their written consent, they were informed that the task consisted of making a cup of tea and a sandwich whilst performing an additional task. The experimenter fitted the headset on the participant and ensured that the eye and corneal reflection were visible and that the scene camera could record fixations in the lower half of the visual field at hand level. A five-point calibration procedure was then conducted by asking each participant to fixate on five toy building bricks distributed across the kitchen counter. In the tea-making task, participants stood up, while in the sandwich-making task, participants were seated. Calibration was performed offline using the software Yarbus (www.positivescience.com), whereby the locations of the building objects were marked up in the scene video frame. The calibration procedure was repeated before each task which helped to correct for drift in eye position across the study.

In the tea-making task, participants were asked to make a single cup of tea using a tea bag and milk. They needed to locate the necessary items (tea bag, spoon and milk), boil a kettle and make the tea. In the sandwich-making task, participants were asked to make a jam sandwich (taking bread from a bag, locating and opening the jam, spreading the jam, and putting the two slices together). For the secondary (load) task, participants were instructed to count backwards starting from 357, either in threes (high-load) or in ones (low-load). If the participant made an error, the experimenter stated ‘incorrect’ and they could then provide the correct number. All participants did both real-world tasks once, one task in the high- and one task in the low-load condition. Assignment of tasks to condition and their order was counterbalanced between participants, with approximately the same number of participants completing each task in each load condition. The study lasted approximately 20 min.

## Results

### Data analysis

Due to problems with eye tracking data loss, data from three participants were excluded. In the remaining participants, gaze data was available for at least 85% of the task duration. We first report behavioural data on task performance (completion time and error rates). Then, we examine the effects of load on gaze to relevant and non-relevant objects. Finally, we look specifically at the eye-hand span which has been previously documented in these tasks.

### Task performance

All participants completed the tasks successfully. The tea task took participants 183.70 s on average (*SD* = 52.30), while the sandwich task was completed more quickly (largely because they did not have to wait for the kettle to boil; *M* = 70.43, *SD* = 32.82). In order to test the effect of load on task completion time (Table 1), we normalised completion times by subtracting the task mean from each time and dividing by the task standard deviation. Normalised times from high- and low-load conditions were then compared using a paired samples t-test. There was a significant difference, *t* (21) = -2.603*, p* = 0.016, with slower completion in the high load condition*.*

**Table 1:** Mean completion time and errors in the counting task (with SD in brackets)

Task	Load	Completion time (s)	Errors
Tea	High	186.65 (39.40)	1.66 (2.10)
	Low	161.4 (35.59)	1.00 (1.09)
Sandwich	High	77.03 (44.20)	0.72 (1.19)
	Low	61.25 (16.77)	0.25 (0.62)

Errors in the counting task, where participants corrected themselves, were rare, with some participants making zero errors (mean number of errors = 1.4 and 0.5 in tea and sandwich tasks, respectively). There were more errors in the high load condition when comparing normalised errors, *t* (1, 21) = -2.152*, p* = 0.043*.*

### Coding of gaze and actions

We analysed gaze behaviour and participants’ actions during the tea and sandwich-making tasks according to the criteria proposed by Land and Hayhoe (2001). Audio–video recordings of the participants’ behaviour captured by the scene camera was hand labelled using Positive Science’s Gaze Tag software. Any fixations that lasted over 100 ms were coded to record gaze on objects of interest.

**Fig. 1:**
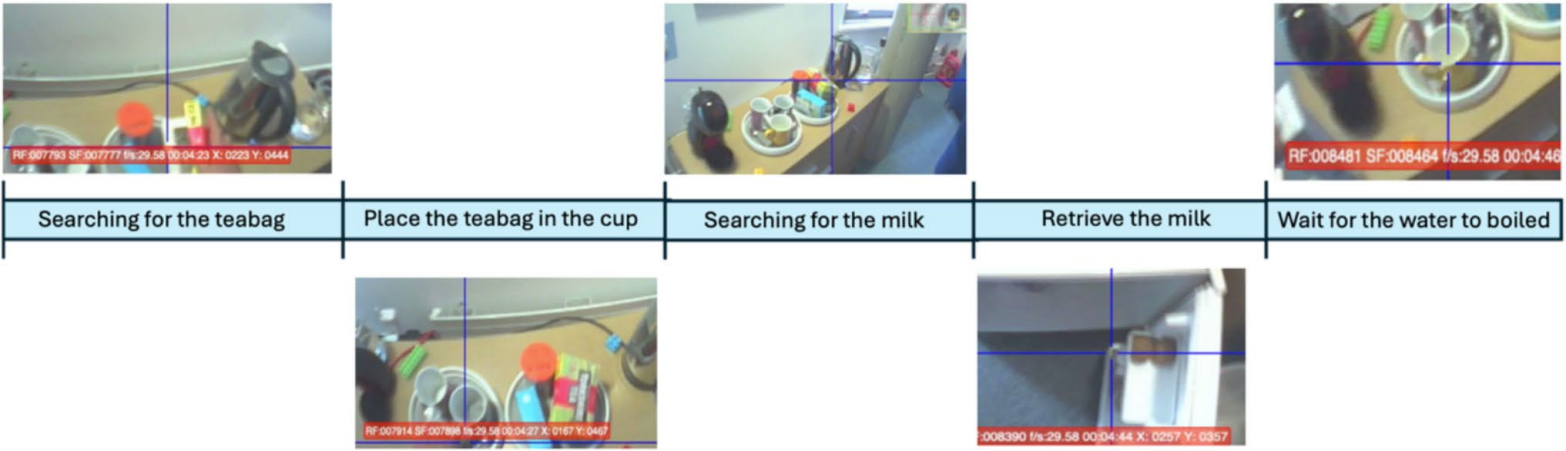
An example of a participant’s fixations during the tea making; representing five ORAs. Each frame shows the scene in front of the participant, with the crosshair indicating the point of gaze

We also coded the object-related actions (ORAs, see Land and Hayhoe 2001) that contribute to achieving the end goal of the task. ORAs are sums of simple actions, as shown in Fig. 1.

**Fig. 2:**
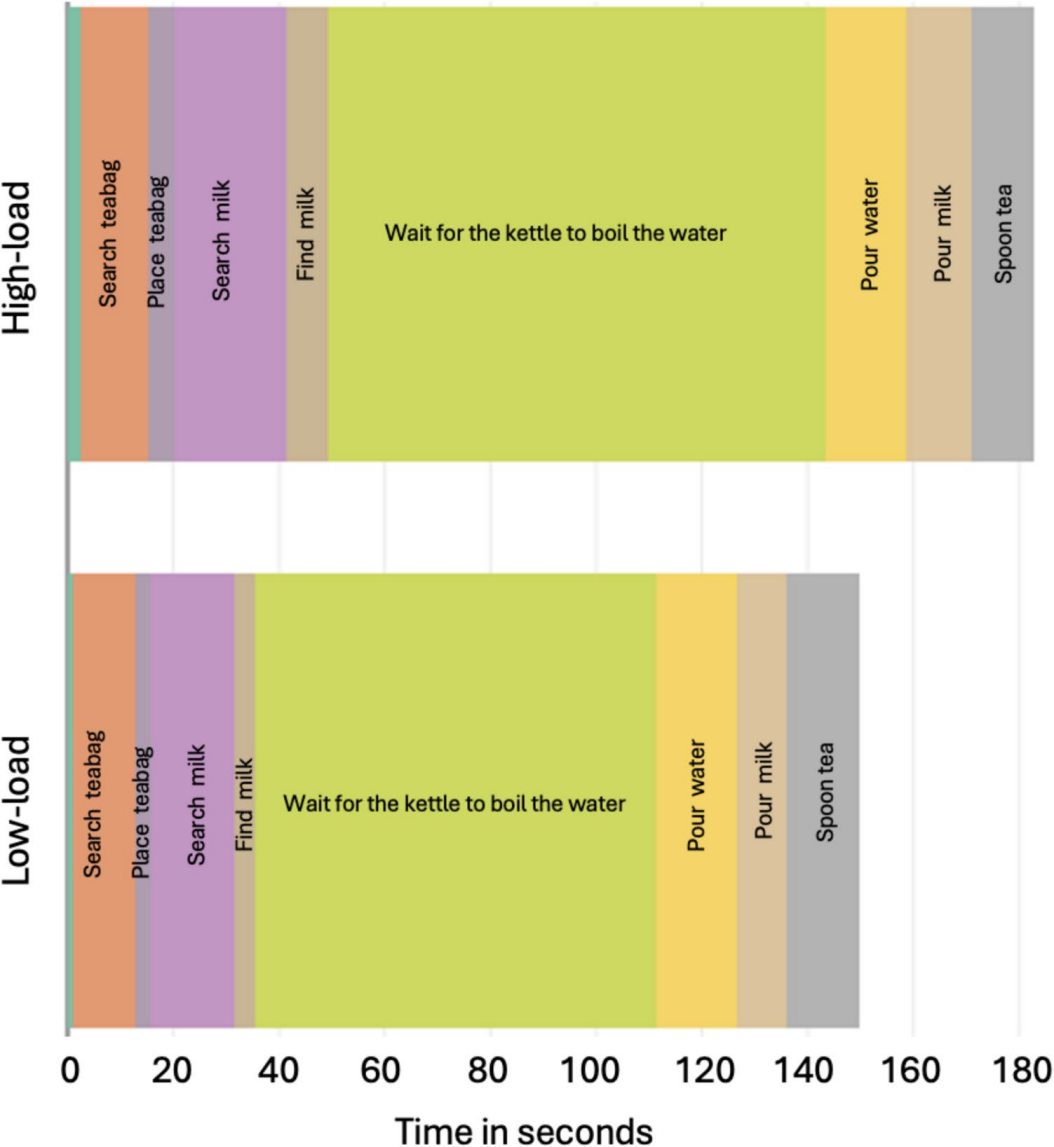
Distribution of time across ORAs in the tea-making task, averaged across participants, in each load condition. The top indicates the high load, and the bottom indicates the low load. The first ORA (turn the kettle on) was very short (2.37 s and 1.13 s, for high and low, respectively)

**Fig. 3:**
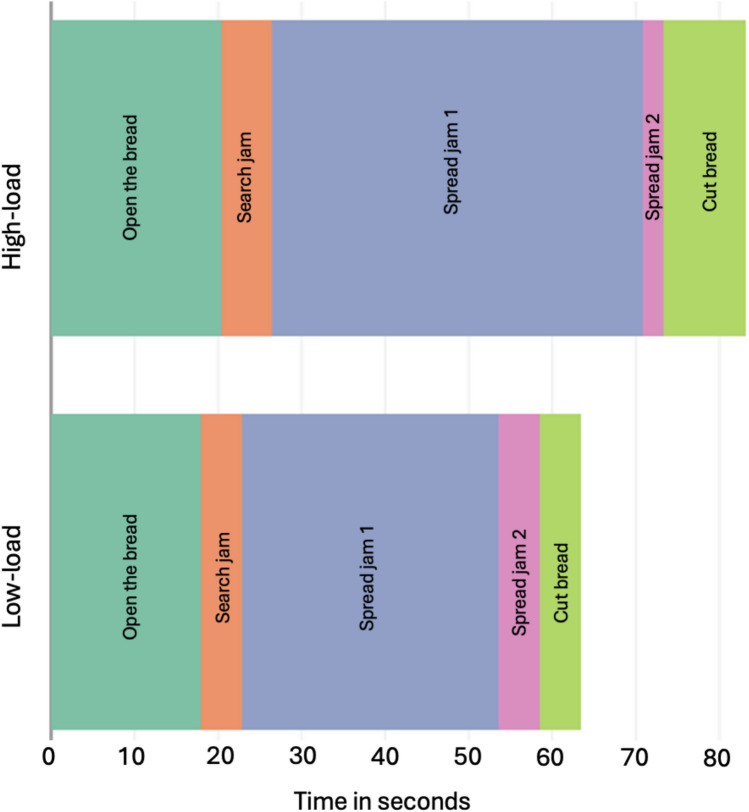
Distribution of time across ORAs in the sandwich task, averaged across participants, in each load condition

Figures 2 and 3 illustrate how the duration of different ORAs varied with the load of the competing task. Overall, participants tended to slow down in the high-load condition, with some ORAs being more affected by load than others, but these differences did not reach statistical significance (all *p* > 0.05). For tea-making, the search behaviour when participants searched for the milk, which involved first locating and opening the fridge, was marginally more prolonged in high vs low load (*p* = *0.05*, although we note this analysis has poor statistical power)*.* Differences in sandwich making (Fig. 3) were also not statistically significant although it is noteworthy that on average the high load condition was particularly prolonged by an extended period of jam-spreading, a rather intricate action involving multiple objects.

### Fixations to objects

For each task, we categorised objects as either relevant or irrelevant. For instance, in the tea-making task, the relevant objects included the kettle and tea bags. In the sandwich-making task, the relevant objects were a loaf of sliced bread, a jar of jam, and a knife. A jar of coffee was irrelevant for both tasks. Other parts of the room such as the walls and ceiling were also coded as irrelevant objects.

**Fig. 4:**
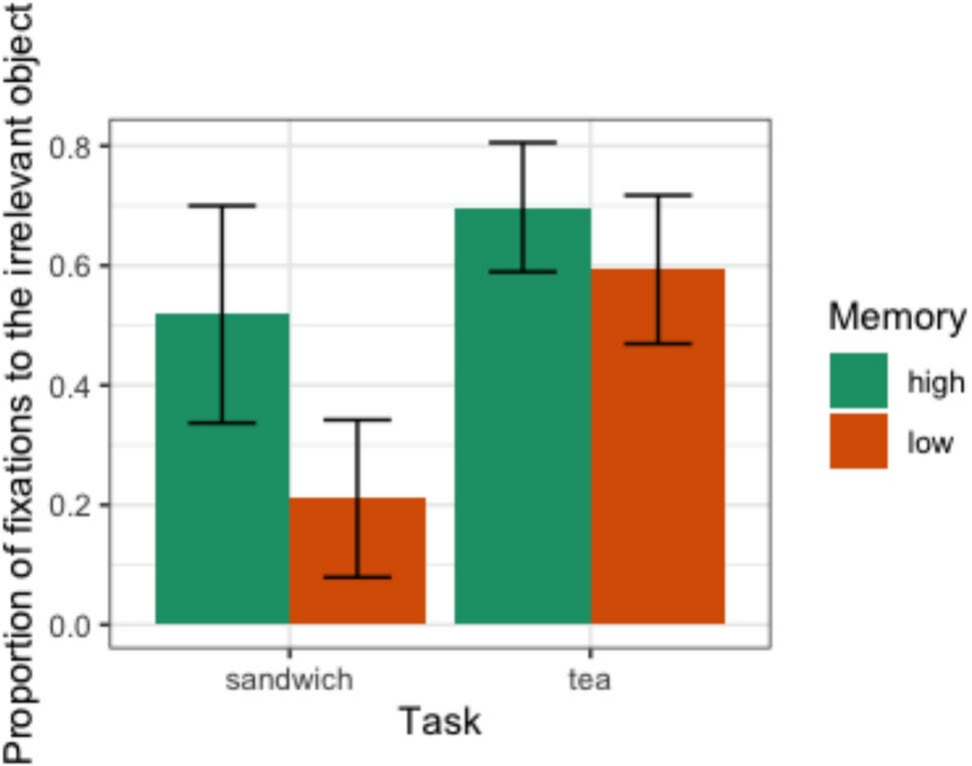
The mean proportion of fixations on irrelevant objects compared to relevant ones across the two tasks. Error bars showing 95% confidence intervals

From the large number of fixations made by each participant and the hand coding of object labels, we calculated the proportion of fixations that occurred on irrelevant versus relevant objects. All fixations were labelled according to object relevance, such that the proportion of fixations on each category added up to 100%, and here we simply analyse how load condition changed this balance. Participants spent more time looking at irrelevant objects under the high-load condition (Fig. 4).

This was confirmed using a generalised linear mixed model (GLMM). We predicted the (binary) relevance of the object being fixated across all fixations (7376 data points), including random effects of participant and task (tea- and sandwich-making), using the lme4 package in R (Bates, Maechler, Bolker, and Walker, 2015) and a binominal function. We then added the fixed effect of load (high or low), which was a significant predictor (maximum likelihood comparison with an intercept-only model: χ^2^(1) = 231.45, *p* < 0.001). The probability of fixating on the irrelevant object under the high-load condition increased relative to the low load condition (β = -1.025 ± 0.070 SE,* p* < 0.001).^1^

### Eye-hand span during tea making

**Fig. 5:**
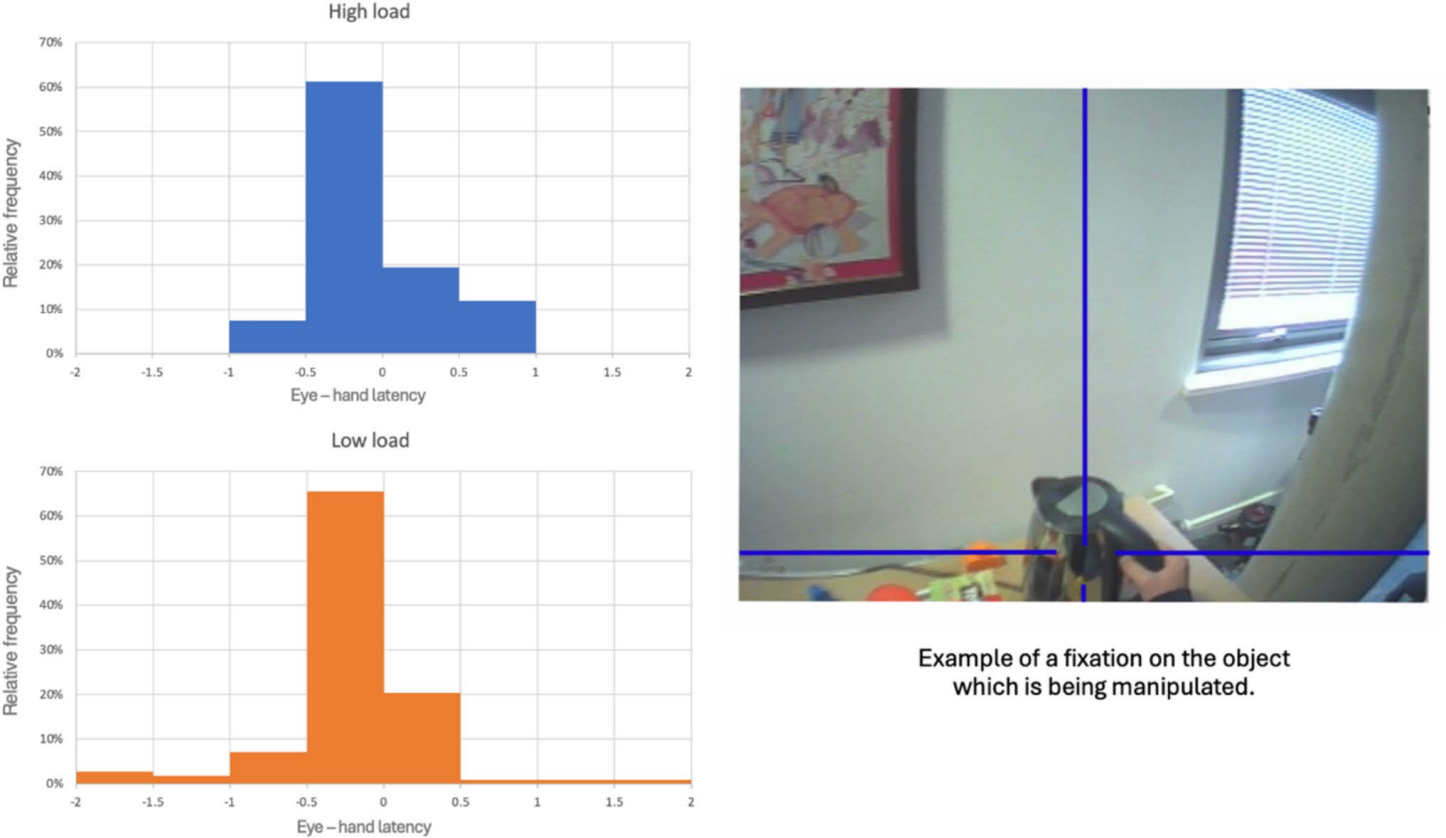
Eye hand latencies, across two actions during tea-making. The x-axis shows the difference between the time at which the manipulation begins (hand touches the object) and the start of the fixation on this object. Negative latencies indicate that the eye moves in advance of the hand

Next, we examined whether the eye-hand span would be affected by load. Our detailed analysis centred around two actions essential for making a cup of tea: pouring boiled water into a cup and adding milk. We chose to focus on these actions because they required manipulating objects and were easily observable from the scene camera. We coded the time the participant touched the object, and compared this to the time when it was fixated. We analysed the data from 10 participants in each condition (due to the angle of the scene camera these events were not observable in the remaining participants). Participants' eyes tended to fixate on each object before any manipulative activity occurred, although this varied depending on the task load *t* (1,9) = 2.454*, p* = 0.024. On average, lead-time was -0.08 s (SD = 0.10) in the high-load condition, and -0.18 s (SD = 0.09) in the low-load. Figure 5 shows the distribution of gaze time relative to the manipulation of an object.

The majority of fixations, in both conditions, precede the hand by about 0.5 s, however, differences in distribution between conditions can also be observed. This is consistent with previous reports (Land and Hayhoe 2001). In the low-load condition, there are few examples where the eye reaches the object after the hand (i.e., with positive eye-hand latencies), and some cases where the eye moves 1–2 s ahead. In the high load condition, this pattern is shifted later in time, such that there is an increase in fixations following, as opposed to leading, the hand.

## Discussion

The present study aimed to investigate the impact of a competing task load on visual attention, specifically gaze patterns, among healthy adult participants engaged in everyday tasks. This naturalistic approach enabled us to assess how increased cognitive demands might influence gaze behaviour during these familiar activities. The findings from this study were as follows.

Firstly, participants were generally slower in high-load conditions, regardless of the task. As expected, they experienced delays while attempting to count backwards in threes at the same time as completing the main task. Previous research has demonstrated that clinical samples experience more errors and longer response times than control groups (Allain et al. 2014; Bieńkiewicz et al. 2015). In this study, we found that response times slowed under high-load conditions in a non-clinical sample. This was also observed by Morady and Humphreys (2010).

The everyday tasks used here were doubtless different in many ways. For example, during tea-making, participants stood up and had to move around more (e.g., to get the milk from the fridge), while in the sandwich-making task, they sat down with all resources within reach. Despite this, we found similarly-sized effects of load in each case. Our description of ORAs provides some hints as to the different behaviours which might be particularly affected by load, although this was limited by statistical power and will require further study. For example, if load particularly affects search behaviour (a component often studied in lab tasks), then we would expect it to prolong some sub-actions (e.g., searching for the milk) more than others (e.g., spreading jam on bread).

A high load competing task also changed the way that fixations were guided towards relevant vs. irrelevant objects. Participants exposed to a concurrent load spent more time looking at irrelevant objects (which are counterproductive for the primary task). This pattern has not, to our knowledge, been reported before. During walking, a cognitively-demanding dual task may cause participants to focus more on the path (Zukowski et al. 2021) or, in other conditions, gaze at the sky (Müller et al. 2023). Previously, it has been reported that looking at irrelevant objects during tea-making is more prevalent in patients with ADS (Forde et al. 2010; Morady and Humphreys, 2010). Our finding aligns with prior research that established the relationship between increased cognitive demands and difficulty managing distractions, such as load theory (Lavie 2010; Lavie et al. 2004). This theory states that when individuals are faced with higher cognitive loads, their ability to filter out irrelevant information is compromised, resulting in a greater susceptibility to distractions (Lavie 2010; Lavie et al. 2004; Martinez-Cedillo et al 2022; 2024). Load theory has been extensively researched in controlled laboratory environments. We extend this beyond highly controlled tasks, which is important at a time when researchers are concerned about the ecological validity of attentional phenomena (Foulsham and Kingstone 2017; Kingstone, et al 2008; Hommel et al 2019). The guidance of gaze in complex tasks, which is rather well-practiced and consistent across tasks and observers, may nonetheless overlap with general processes in visuospatial attention such as distractor rejection, and rely on cognitive resources which are taxed in verbal memory load paradigms. Of course, at a neurophysiological level, the interference that we have demonstrated between attentional resources and eye movements is not surprising. Modern research has shown that neural control of eye movements is intertwined with more general attentional mechanisms. For example, Noudoost, Clark and Moore (2021) report that when cognitive control is engaged in macaques, visual input to the frontal eye fields (FEF) becomes stronger, faster and more synchronised, suggesting that the ability to focus and prioritise sensory information is dynamically modulated by cognitive control.

There are two not-unrelated explanations for why participants in high-load conditions might have looked more at irrelevant objects. The first is that it may have been a deliberate strategy for managing the competing tasks, in which participants looked away while engaged in the counting task. Gaze aversion has previously been observed in conversation, where looking away from a partner can help manage the load of thinking about what to say (Doherty-Sneddon and Phelps 2005). Müller et al. (2023) suggest that raised gaze when walking with a dual task might occur to reduce visual input (and focus on the cognitive task at hand). The second is that top-down commands, guiding the gaze system to look at the necessary objects for the task, were disrupted by cognitive load. Land (2009) proposes a “schema system” which provides such commands during manual tasks and which influences where people look and the information extracted. In our study it seems that the more difficult load task meant that some of the time these top-down commands were delayed or disrupted, such that other objects captured attention instead (perhaps because they were salient in a bottom-up sense). Future studies could manipulate the saliency of these distractors as a way of distinguishing between these explanations.

A third finding of interest was that we observed a significant change in the way that the eyes and the hands were co-ordinated. In most cases, the eyes fixated an object a short time before the hand movement which contacted it (e.g., when picking up the kettle). This pattern has been previously observed. Land et al (1999) reported a mean lag between eye and hand of 0.56 s from 3 participants making tea. In more recent analysis of 24 participants who were eye and motion tracked during object manipulation tasks, Lavoie et al. (2018) report average lags of between 0.5 and 0.7 s. The exact lag observed probably depends on the distances and objects involved (Hayhoe et al. 2003, report a shorter average lag during sandwich making). Importantly, in our study, this latency was affected by cognitive load. Participants in the high load condition were less likely to look to the target object in advance of reaching for it, and in a significant minority of actions they reached for it without a prior fixation (i.e., the eye followed the hand rather than the other way around). This may suggest that the rather systematic timing of gaze in such tasks is not “attention-free” and is therefore disrupted by load. Interestingly, participants were still able to contact and begin the action even without visual supervision, which may mean that they are compensating for the load task (by fixating elsewhere while the hand proceeds). The present results indicate that future studies investigating which components of eye-hand coordination are disrupted by cognitive load would be fruitful.

It is important to note that previous studies with artificial, computer-based tasks have indicated that the type of load task may change the interference suffered. For example, Burnham et al., (2014) found that counting backwards (a similar task to the one we used here) increased singleton interference in an attentional capture task, while a phonological working memory task did not. Different types of load task might also go some way to explaining the inconsistent results regarding dual tasks and gaze during walking (Zukowski et al. 2021; Walsh and Snowball 2023; Müller et al. 2023). In complex scene viewing, we have previously found larger interference effects from a cognitive load task involving spatial memory (remembering a pattern of dots) than from one involving verbal memory (remembering a number; Martinez-Cedillo et al. 2022, 2024). It is possible that a load which was more closely related to the primary task, or which involved the spatial locations characteristic to making tea or sandwiches, would have had more impact. According to Baddeley’s working memory model (1996), counting backwards would involve the central executive and processes which are required for monitoring ongoing actions and goals in the tasks used here (including the control of gaze by Land’s 2009 schema system). It remains to be determined whether a working memory task which involved only the maintenance of visual or spatial information would also disrupt gaze or actions in these tasks.

Our study aimed to investigate the impact of varying levels of cognitive load on daily tasks. Previous research (e.g., Lavie et al. 2004) suggested that increased cognitive load can impair task performance, reducing efficiency and increasing distraction. A possible limitation of the current study is that we examined only low- and high- load conditions, rather than comparing these to a single-task (“no load”) baseline which has sometimes been used in prior work with computer-based tasks (e.g., Burnham et al. 2014). Comparing a single-task baseline in the same study would be a useful addition in future research. This design does however indicate that the differences in eye movements that we observe here are not due to a strategic difference in scheduling two tasks at the same time, but rather to the actual difficulty of the dual task. The presence of a distractor task, even in the low load condition, may explain why we observed fixations on irrelevant objects which were more common than in previous reports of natural tasks performed without any concurrent load (e.g., Land and Hayhoe 2001).

Another possible limitation is that we did not consider individual differences in working memory capacity. We observed minimal individual differences in the completion and errors in the counting task. However, we do not rule out the possibility that individual differences play a significant role in the amount of interference experienced while performing everyday tasks. Research by Unsworth and Engle (2007) and Conway and Engle (1996) shows that individuals with high working memory capacity excel in high-load tasks due to higher attentional control and efficient retrieval. We therefore predict that such individuals would show less affected eye movements in the presence of a dual task. Future studies could investigate individual variations in working memory capacity as a factor involved in gaze during realistic tasks.

## Conclusion

This study explored gaze behaviour in high versus low load conditions while participants engaged in everyday tasks, such as making tea and sandwiches. The findings revealed that under high load conditions, participants were slower and had more fixations on irrelevant objects, compromising their ability to filter out distractions. Furthermore, eye and hand coordination was also affected by high load, with participants being less likely to move their eyes in advance of their hand. These findings begin to shed light on the control of visual attention in naturalistic tasks and the cognitive resources involved.

## Data Availability

We have shared the link to our data/code in the manuscript. The data and materials for the experiments are available at: https://osf.io/6qnwj/?view_only=8d16cc94859845b89f8370a13219f7c.

## Footnotes

1 The tea-making task included a prolonged period in which participants were waiting for the kettle to boil. Since participants might let their gaze wander more freely at this time, we repeated the analysis of fixations on irrelevant objects, but excluding this waiting period. The increase in looks to irrelevant objects under high cognitive load remained. We thank an anonymous reviewer for this suggestion.

## References

[CR1] Allain P, Foloppe DA, Besnard J, Yamaguchi T, Etcharry-Bouyx F, Le Gall D, Nolin P, Richard P (2014) Detecting everyday action deficits in Alzheimer’s disease using a nonimmersive virtual reality kitchen. J Int Neuropsychol Soc 20(5):468–477 24785240 10.1017/S1355617714000344

[CR2] Baddeley A (1996) The fractionation of working memory. Proc Natl Acad Sci 93(24):13468–13472 8942958 10.1073/pnas.93.24.13468 PMC33632

[CR3] Ballard DH, Hayhoe MM (2009) Modelling the role of task in the control of gaze. Vis Cogn 17(6–7):1185–1204 20411027 10.1080/13506280902978477 PMC2856937

[CR4] Bates D, Maechler M, Bolker B, Walker S, Christensen RHB, Singmann H, Bolker MB (2015) Package ‘lme4.’ Convergence 12(1):2

[CR5] Bieńkiewicz MM, Brandi ML, Hughes C, Voitl A, Hermsdörfer J (2015) The complexity of the relationship between neuropsychological deficits and impairment in everyday tasks after stroke. Brain and Behavior 5(10):e00371 26516606 10.1002/brb3.371 PMC4614052

[CR6] Boot WR, Kramer AF, Peterson MS (2005) Oculomotor consequences of abrupt object onsets and offsets: onsets dominate oculomotor capture. Percept Psychophys 67:910–928 16334062 10.3758/bf03193543

[CR7] Burnham BR, Sabia M, Langan C (2014) Components of working memory and visual selective attention. J Exp Psychol Hum Percept Perform 40(1):391 23875574 10.1037/a0033753

[CR8] Conway ARA, Engle RW (1996) Individual differences in working memory capacity: more evidence for a general capacity theory. Memory 4(6):577–590. 10.1080/741940997 8934455 10.1080/741940997

[CR9] Doherty-Sneddon G, Phelps FG (2005) Gaze aversion: a response to cognitive or social difficulty? Mem Cognit 33:727–733 16248336 10.3758/bf03195338

[CR10] Forde EM, Humphreys GW (2000) The role of semantic knowledge and working memory in everyday tasks. Brain Cogn 44(2):214–252 11041990 10.1006/brcg.2000.1229

[CR11] Forde EME, Rusted J, Mennie N, Land M, Humphreys GW (2010) The eyes have it: an exploration of eye movements in action disorganisation syndrome. Neuropsychologia 48(7):1895–1900 20171234 10.1016/j.neuropsychologia.2010.01.024

[CR12] Foulsham T (2015) Eye movements and their functions in everyday tasks. Eye 29(2):196–199 25397783 10.1038/eye.2014.275 PMC4330286

[CR13] Foulsham T, Kingstone A (2017) Are fixations in static natural scenes a useful predictor of attention in the real world? Canad J Exp Psychol 71(2):172 28604053 10.1037/cep0000125

[CR202] Hayhoe MM, Shrivastava A, Mruczek R, Pelz JB (2003) Visual memory and motor planning in a natural task. J Vis 3(1):49–63. 12678625 10.1167/3.1.6

[CR16] Hayhoe MM, McKinney T, Chajka K, Pelz JB (2012) Predictive eye movements in natural vision. Exp Brain Res 217:125–136 22183755 10.1007/s00221-011-2979-2 PMC3328199

[CR18] Hommel B, Chapman CS, Cisek P, Neyedli HF, Song JH, Welsh TN (2019) No one knows what attention is. Atten Percept Psychophys 81:2288–2303 31489566 10.3758/s13414-019-01846-w PMC6848248

[CR19] Jovancevic J, Sullivan B, Hayhoe M (2006) Control of attention and gaze in complex environments. J vis 6(12):9–9 10.1167/6.12.9 17209746

[CR20] Kingstone A, Smilek D, Eastwood JD (2008) Cognitive ethology: a new approach for studying human cognition. Br J Psychol 99(3):317–340 17977481 10.1348/000712607X251243

[CR21] Kothari R, Yang Z, Kanan C, Bailey R, Pelz JB, Diaz GJ (2020) Gaze-in-wild: A dataset for studying eye and head coordination in everyday activities. Sci Rep 10(1):2539 32054884 10.1038/s41598-020-59251-5 PMC7018838

[CR22] Lamers MJ, Roelofs A (2011) Attention and gaze shifting in dual-task and go/no-go performance with vocal responding. Acta Physiol (Oxf) 137(3):261–268 10.1016/j.actpsy.2010.12.005 21549333

[CR23] Land MF (2009) Vision, eye movements, and natural behavior. Vis Neurosci 26(1):51–62 19203425 10.1017/S0952523808080899

[CR24] Land MF, Hayhoe M (2001) In what ways do eye movements contribute to everyday activities? Vision Res 41(25–26):3559–3565 11718795 10.1016/s0042-6989(01)00102-x

[CR25] Land M, Tatler B (2009) Looking and acting: vision and eye movements in natural behaviour. Oxford University Press, USA

[CR26] Land M, Mennie N, Rusted J (1999) The roles of vision and eye movements in the control of activities of daily living. Perception 28(11):1311–1328 10755142 10.1068/p2935

[CR27] Lavie N (2005) Distracted and confused?: selective attention under load. Trends Cogn Sci 9(2):75–82 15668100 10.1016/j.tics.2004.12.004

[CR28] Lavie N (2010) Attention, distraction, and cognitive control under load. Curr Dir Psychol Sci 19(3):143–148

[CR29] Lavie N, Hirst A, De Fockert JW, Viding E (2004) Load theory of selective attention and cognitive control. J Exp Psychol Gen 133(3):339 15355143 10.1037/0096-3445.133.3.339

[CR30] Lavoie EB, Valevicius AM, Boser QA, Kovic O, Vette AH, Pilarski PM, Chapman CS (2018) Using synchronized eye and motion tracking to determine high-precision eye-movement patterns during object-interaction tasks. J Vision 18(6):18–18 10.1167/18.6.18 30029228

[CR33] Martinez-Cedillo AP, Dent K, Foulsham T (2022) Do cognitive load and ADHD traits affect the tendency to prioritise social information in scenes? Quart J Exp Psychol 75(10):1904–1918 10.1177/17470218211066475 PMC9424720 34844477

[CR34] Martinez-Cedillo AP, Dent K, Foulsham T (2024) Social prioritisation in scene viewing and the effects of a spatial memory load. Atten Percept Psychophys 86(4):1237–1247 37563513 10.3758/s13414-023-02769-3 PMC11093800

[CR35] Matthis JS, Yates JL, Hayhoe MM (2018) Gaze and the control of foot placement when walking in natural terrain. Curr Biol 28(8):1224–1233 29657116 10.1016/j.cub.2018.03.008 PMC5937949

[CR36] Matthis JS, Muller KS, Bonnen KL, Hayhoe MM (2022) Retinal optic flow during natural locomotion. PLoS Comput Biol 18(2):e1009575 35192614 10.1371/journal.pcbi.1009575 PMC8896712

[CR200] Morady K, Humphreys GW (2009) Comparing action disorganization syndrome and dual-task load on normal performance in everyday action tasks. Neurocase 15(1):1-12 10.1080/13554790802524214 19065284

[CR201] Morady K, Humphreys G (2011) Multiple task demands in action disorganization syndrome. Neurocase 17(5):461–472 21506044 10.1080/13554794.2010.532144

[CR39] Müller C, Baumann T, Einhäuser W, Kopiske K (2023) Slipping while counting: gaze–gait interactions during perturbed walking under dual-task conditions. Exp Brain Res 241(3):765–780 36725725 10.1007/s00221-023-06560-6 PMC9985588

[CR40] Noudoost B, Clark KL, Moore T (2021) Working memory gates visual input to primate prefrontal neurons. Elife 10:e64814. 10.7554/eLife.64814 34133270 10.7554/eLife.64814 PMC8208812

[CR43] Schwartz MF, Reed ES, Montgomery M, Palmer C, Mayer NH (1991) The quantitative description of action disorganisation after brain damage: a case study. Cogn Neuropsychol 8(5):381–414

[CR44] Sullivan B, Ludwig CJ, Damen D, Mayol-Cuevas W, Gilchrist ID (2021) Look-ahead fixations during visuomotor behavior: evidence from assembling a camping tent. J vis 21(3):13–13 33688920 10.1167/jov.21.3.13 PMC7961111

[CR45] Unsworth N, Engle RW (2007) The nature of individual differences in working memory capacity: active maintenance in primary memory and controlled search from secondary memory. Psychol Rev 114(1):104–132. 10.1037/0033-295X.114.1.104 17227183 10.1037/0033-295X.114.1.104

[CR46] Walsh GS, Snowball J (2023) Cognitive and visual task effects on gaze behaviour and gait of younger and older adults. Exp Brain Res 241(6):1623–1631 37148282 10.1007/s00221-023-06627-4 PMC10224856

[CR47] Ward G, Morris R (2004) Introduction to the psychology of planning. The cognitive psychology of planning. Psychology Press, pp 11–13

[CR48] Zukowski LA, Tennant JE, Iyigun G, Giuliani CA, Plummer P (2021) Dual-tasking impacts gait, cognitive performance, and gaze behavior during walking in a real-world environment in older adult fallers and non-fallers. Exp Gerontol 150:111342 33838215 10.1016/j.exger.2021.111342 PMC8164995

